# Multiscale entropy rate analysis of complex mobile agents

**DOI:** 10.1098/rsos.180488

**Published:** 2018-10-10

**Authors:** Tuhin Paul, Kevin G. Stanley, Nathaniel D. Osgood

**Affiliations:** Department of Computer Science, University of Saskatchewan, Saskatoon, Saskatchewan, Canada

**Keywords:** human mobility model, mobility entropy rate, Lempel–Ziv entropy

## Abstract

Accurate prediction of the motion of objects is a central scientific goal. For deterministic or stochastic processes, models exist which characterize motion with a high degree of reliability. For complex systems, or those where objects have a degree of agency, characterizing motion is far more challenging. The information entropy rate of motion through a discrete space can place a limit on the predictability of even the most complex or history-dependent actor, but the variability in measured encountered locations is inexorably tied to the spatial and temporal resolutions of those measurements. This relation depends on the path of the actor in ways that can be used to derive a general law in closed form relating the mobility entropy rate to different spatial and temporal resolutions, and the path properties within each cell along the path. Correcting for spatial and temporal effects through regression yields the path properties and a measure of mobility entropy rate robust to changes in dimension, allowing comparison of mobility entropy rates between datasets. Employing this measure on empirical datasets yields novel findings, from the similarity of taxicabs to drifters, to the predictable motions of undergraduates, to the browsing habits of Canadian moose.

## 1. Introduction

How predictable is the motion of an object though space? For many deterministic or stochastic systems, this question can be answered with a degree of certainty using well-established models. However, for complex physical systems, or systems where the actors have a degree of agency, this question remains difficult to answer. In their seminal work, Song *et al.* demonstrated how the measured entropy rate of a person moving through discretized space could be used to estimate the asymptotic predictability of human actors [1]. While their analysis focused on the daily habits of individuals, their analysis technique is generally applicable to any object moving through a discretized space. However, their core conclusion—that human mobility is inherently predictable—was only established for the dataset that they considered: predominantly urban dwellers who own and use cellular phones on a regular basis. Because of its reliance on cellular call records, as in subsequent works [2–4], their dataset was subject to bias both by its focus on a particular demographic, and adherence to a particular spatial and temporal resolution. More recent empirical research has established that the estimated predictability of human mobility is contingent on the scale [5] and structure [6,7] of the data, and underlying mobility model assumptions [8]. While Song *et al.* made a foundational contribution to quantifying mobility predictability in complex systems, their results are only applicable to the population and spatio-temporal resolution [8] represented in the data.

Because the mobility entropy rate depends on both the spatio-temporal resolution of the collected data, and the properties of the path, comparisons of entropy rate between experiments or agents captured with different apparatus cannot be meaningfully conducted. If the combination of these properties is deterministic, then a model could be derived which separated the system error (spatio-temporal resolution) from the observation (path properties). This paper builds upon the results of [8] to derive a general solution to the scaling of mobility entropy rate estimates in a discretized space, based on observations about the structure of paths through a discrete grid. The model shows considerable agreement with empirical data for agents as diverse as university students, taxicabs, moose and ocean buoys. Employing the scaling relationship allows researchers to analyse the predictability of the mobility traces at spatial scales consistent with the underlying mobility, to renormalize mobility data to common spatio-temporal resolutions for meta-analysis, to predict the impact of increasing or decreasing the spatio-temporal resolution of their study, and—through the analysis of the scaling relationship parameters—come to actionable conclusions about the relative mobility behaviours of individuals or populations.

## 2. Material and methods

Song *et al.*’s central methodological insight [1]—that an agent’s passage through a discretized space creates a sequence of symbols corresponding to the locations traversed (a visit string)—constituted a foundational contribution. Like all strings, the visit string has an intrinsic information content which can be readily approximated using Lempel–Ziv compression. As string length tends towards infinity, the compressibility of the string tends towards the information entropy rate of the string, which describes the limit of the string’s predictability [9]. However, different spatial and temporal resolutions will create different symbolic representations of the same trajectory (e.g. doubling spatial bin size will re-render the string AABBCCDD as AAAACCCC) and, therefore, lead to different intrinsic entropy rate estimates for each spatio-temporal representation. The entropy rate of a visit string, *S*, can be estimated as

2.1 $$H={\left(\frac{1}{L}\sum_{i=0}^{L-1}\Lambda_{i}\right)}^{-1}\log L,$$

where *Λ*_*i*_ is the length of the shortest substring of *S* starting at position *i* that has not previously been encountered, and *L* is the overall length of *S* [1–4]. In [8], a theoretical framework inspired by the spatio-temporal effects observed in other studies [5,7] was formulated that relates the entropy rate from Song *et al.*’s approach to the scale of the spatial binning and temporal sampling for constrained paths. Key assumptions were required to split the summation in (2.1) into *n* distinct substrings, which limited the applicability of the findings.

When considering paths which traverse implicitly (due to accuracy limitations) or explicitly (due to experimental structure) discretized spaces and times, the discretization itself can be leveraged to relax previous assumptions. Actors with agency can traverse a cell along a nonlinear path, and potentially stop along the way, leading to

2.2 $$t_{i}=\frac{d}{v_{i}^{*}}+t_{d_{i}},$$

where *t*_*i*_ is the time to traverse the *i*th cell, *d* is the width of the cell, v*_i_ is the apparent velocity across the cell, and *t*_*d*_*i*__ is the total (stationary) dwell time within the cell. The apparent velocity across the cell is the average velocity, while moving, across the length of the cell and incorporates the actual average velocity, and the path length through the cell. The scaling behaviour of the entropy rate can be estimated by examining the kinds of sequences created as the agent traverses cells.

Let the spatio-temporal resolution be represented as a tuple (*T*, *d*), where *T* is the sampling interval and *d* is the side length of a square cell in the spatial grid. We assume square cells for simplicity in spatial quantization, and define *d* as the length of an edge, or the characteristic length of a cell.

The time spent while in motion in the *i*th cell is expressed as *t*_*m*_*i*__. Without considering the dwell time, the apparent average velocity in the *i*th cell along the path of an agent is shown in

2.3 $$v_{i}^{*}=\frac{d}{t_{m_{i}}}.$$

The total time spent in the *i*th cell is the sum of the time spent in motion and the dwell time. The time spent in motion inside the *i*th cell is *d*/v*_i_ (from (2.3)). The total time spent in the *i*th cell is, therefore, expressed as

2.4 $$\begin{array}{rl} t_{i} & =t_{m_{i}}+t_{d_{i}}\\ & =\frac{d}{v_{i}^{*}}+t_{d_{i}} \end{array}$$

The total dwell time in the *i*th cell is the sum of dwells in the *i*th cell, as shown in

2.5 $$t_{d_{i}}=\sum_{k}^{}t_{d_{i}}^{k}.$$

Therefore, the observable average velocity considering dwell time, ${\tilde{v}}_{i}$, while passing the *i*th cell, can be expressed as

2.6 $${\tilde{v}}_{i}=\frac{d}{t_{i}}=\frac{d}{d/v_{i}^{*}+t_{d_{i}}}.$$

Let the agent travel through *n* cells on its entire path, treating repetitions of a cell separately. The number of blocks of repeating strings along the path would be represented by *n*. The total time spent in motion on the entire path is expressed as *t*_*m*_, which is the sum of each *t*_*m*_*i*__, as shown in

2.7 $$t_{m}=\sum_{i=1}^{n}t_{m_{i}}.$$

Considering only motion, and *nd* as the total traversed length, the apparent average velocity v* is shown in

2.8 $$v^{*}=\frac{nd}{t_{m}}.$$

The total dwell time along the entire path is the summation of dwell times in each cell, as shown in

2.9 $$t_{d}=\sum_{i=1}^{n}t_{d_{i}}$$

The observable average velocity for the entire path, considering dwell times as well, is represented as $\tilde{v}$, as shown in

2.10 $$\begin{array}{rl} \tilde{v} & =\frac{nd}{t_{1}+t_{2}+\cdots +t_{n}}\\ & =\frac{nd}{\left(t_{m_{1}}+t_{d_{1}})+(t_{m_{2}}+t_{d_{2}})+\cdots +(t_{m_{n}}+t_{d_{n}}\right)}\\ & =\frac{nd}{\sum_{i=1}^{n}t_{m_{i}}+\sum_{i=1}^{n}t_{d_{i}}} \end{array}$$

From (2.7), we find that $\sum_{i=1}^{n}t_{m_{i}}=t_{m}$. From (2.8), we find that *t*_*m*_ = *nd*/v*. Substituting them into (2.10), we find $\tilde{v}$ as shown in

2.11 $$\begin{array}{rl} \tilde{v} & =\frac{nd}{\sum_{i=1}^{n}t_{m_{i}}+\sum_{i=1}^{n}t_{d_{i}}}\\ & =\frac{nd}{t_{m}+t_{d}}\\ & =\frac{nd}{nd/v^{*}+t_{d}}=\frac{d}{d/v^{*}+t_{d}/n}. \end{array}$$

There are *n* blocks along the entire path. Let *L*_*i*_ represent the length of the *i*th block, and *L* be the length of the entire string. For simplicity, we assume that *L*_*i*_ is an even integer, and is approximated as

2.12 $$L_{i}=\frac{t_{i}}{T}=\frac{1}{T}\left(\frac{d}{v_{i}^{*}}+t_{d_{i}}\right).$$

Similarly, *L* can be expressed as

2.13 $$L=\frac{t}{T}=\frac{1}{T}(t_{m}+t_{d}).$$

Assuming a unique terminating symbol at the end of the string representing the path, and using the same decomposition as the theoretical model [8], we can express $\sum_{i=0}^{L-1}\Lambda_{i}$ in (2.1) as

2.14 $$\begin{array}{rl} \sum_{i=0}^{L-1}\Lambda_{i} & =\sum_{i=1}^{n}\sum_{\;j=1}^{L_{i}}\Lambda_{j}\\ & =\sum_{i=1}^{n}\left[2\sum_{\;j=1}^{L_{i}/2}j+\frac{L_{i}}{2}\right]\\ & =\sum_{i=1}^{n}\left[\frac{L_{i}^{2}}{4}+L_{i}\right]. \end{array}$$

Substituting (2.12) into (2.14), we can express $\sum_{i=0}^{L-1}\Lambda_{i}$ as

2.15 $$\begin{array}{rl} \sum_{i=0}^{L-1}\Lambda_{i} & =\sum_{i=1}^{n}\left[\frac{((1/T){\left(d/v_{i}^{*}+t_{d_{i}})\right)}^{2}}{4}+\frac{1}{T}\left(\frac{d}{v_{i}^{*}}+t_{d_{i}}\right)\right]\\ & =\frac{1}{4T^{2}}\sum_{i=1}^{n}\left[{\left(\frac{d}{v_{i}^{*}}+t_{d_{i}}\right)}^{2}+4T\left(\frac{d}{v_{i}^{*}}+t_{d_{i}}\right)\right]\\ & =\frac{1}{4T^{2}}\sum_{i=1}^{n}\left[d^{2}\frac{1}{{v_{i}^{*}}^{2}}+t_{d_{i}}^{2}+2d\frac{t_{d_{i}}}{v_{i}^{*}}+4dT\frac{1}{v_{i}^{*}}+4Tt_{d_{i}}\right]\\ & =\frac{1}{4T^{2}}\left(d^{2}\sum_{i=1}^{n}\frac{1}{{v_{i}^{*}}^{2}}+\sum_{i=1}^{n}t_{d_{i}}^{2}+2d\sum_{i=1}^{n}\frac{t_{d_{i}}}{v_{i}^{*}}+4dT\sum_{i=1}^{n}\frac{1}{v_{i}^{*}}+4T\sum_{i=1}^{n}t_{d_{i}}\right). \end{array}$$

Denoting the sampling period as *T*, substituting *t*_*i*_/*T* for *L*_*i*_, substituting into the formula for *H*, and regrouping into distinct terms in the denominator, we obtain (see the electronic supplementary material for derivation)

2.16 $$H(d,T)=\frac{\log L}{\begin{array}{c} \left(d^{2}/4LT^{2})\sum_{i=1}^{n}(1/{v_{i}^{*}}^{2})+(1/4LT^{2})\sum_{i=1}^{n}t_{d_{i}}^{2}+(2d/4LT^{2})\sum_{i=1}^{n}(t_{d_{i}}/v_{i}^{*}\right)\\ +(4dT/4LT^{2})\sum_{i=1}^{n}(1/v_{i}^{*})+(4T/4LT^{2})\sum_{i=1}^{n}t_{d_{i}} \end{array}},$$

where *L* is the length of the string, and *d* is the cell size. The numerator of this scaling relationship is the entropy of a string of unique symbols of length *L*, and the denominator is the amount by which that value is scaled. This scaling reflects both intrinsic characteristics of the path such as dwell time and speed of traversal as well as features of the sampling regime such as cell size and the sampling rate. Specifically, the sums aggregate terms involving v*_i_ and *t*_*d*_*i*__, are the intrinsic mobility parameters of the agent or object, which impact the *L*_*i*_ according to (2.12). Note that each term in the denominator corresponds to a number of samples or symbols in the string implicitly summed over a block to obtain *L*_*i*_, and explicitly summed over all *n* blocks, and represent the impact of that property across the path. When divided by *L*, these values represent an average impact of that particular term over the entire visit string. If we consider v*_i_, *t*_*d*_*i*__ and their squares to be well-behaved random variables with means that are broadly invariant under resampling, then the overall average should converge, independent of *d* and *T* [10]. If there is inherent scaling behaviour within the data generating mobility process underlying a dataset, where specific phases of motion are evident at particular scales, this approximation would only be valid for a single regime, and the more complex functional dependence of v*_i_ and *t*_*d*_*i*__ on *d* and *T* must be empirically approximated (see electronic supplementary material).

Additional understanding of the dependence of *n*, v*_i_ and *t*_*d*_*i*__ on bin size *d* and sampling period *T* can be obtained by examining sampling regimes at the limits of sampling. When *T* is small enough to ensure that all traversed cells are captured (oversampling in *T*), then *n* is independent of *T* because changing *T* will change the size of the block of symbols, not the number of blocks. By contrast, changing *d* will in general change *n*, and in turn change v*_i_ and *t*_*d*_*i*__. For a very small bin size *d*, and consequently large *n* (oversampling in *d*), the summation approximates a spatial integral of a function of v*_i_ or *t*_*d*_*i*__. As long as the bin sizes are small compared with the variability of the function in v*_i_ or *t*_*d*_*i*__, and the function is locally smooth, the integral should not be impacted. Once bin size increases to the point that the summation and continuous integral diverge, (2.16) no longer holds. For regimes where *T* is sufficiently large to skip cells on the path, *n* also depends on *T*. We expect most natural paths and sensible measurement regimes to provide sufficient smooth paths and fine-grained *d* and *T*; however, degenerate paths could be imagined. When our scaling relationship departs from empirical values, it indicates that the sampling regime may not be appropriate for observed paths.

To validate our model, we computed the entropy scaling behaviour of six distinct datasets comprising human, animal and complex physical systems. We have not considered cellphone tower-based datasets, because their lack of spatial and temporal resolution and regularity could confound the model in its current form.

*Saskatchewan Human Ethology Dataset* (*SHED*) *7 and 8*: GPS/WiFi based location records from smartphone-based data collection over a four-week period of 63 and 75 university-affiliated participants, primarily undergraduates, in the summer and autumn of 2016 in a mid-sized Canadian city. Participants who returned at least 15 records for each 8 h interval were retained.
*Roman Taxis*: Over 21 million GPS records of locations at approximately 7 s intervals, publicly available in the Crawdad repository [11]. Over 350 000 records from the top 59 taxi drivers, who returned at least 15 records at 8 h sampling intervals, based on number of reported records, were retained.
*Moose*: in study area of south-central Saskatchewan, telemetered with GPS tracking collars [12]. Moose tend to live solitary nomadic lives, browsing and sleeping at their pleasure over a home range. All 36 moose were considered.
*Antarctic petrel*: movements characterized by 55 176 GPS traces of petrel behaviour [13]. Petrels graze the surface of the water for small fish and crustaceans, only occasionally returning to their nesting sites during breeding season. All 124 petrels were considered.
*Buoys*: in the Juan de Fuca Strait. Nine drifters were released off the coast of Vancouver, Canada to study the impact of surface and tidal currents in distributing pollution generated by the city and port [14]. As buoys have no agency, they follow paths dictated by tides, wind and currents. All nine buoys were considered.

Bounding boxes were used in some cases to limit the scope of some datasets. The bounding box is either computed from the minimum and maximum latitude and longitude values found in the data or arbitrarily from the expected span of the participants’ movements in the study (e.g. a city). The minimum/maximum longitude/latitude values are presented in table 1.

**Table 1.** Longitude and latitude bounds for filtering GPS traces.

dataset	longitude (min)	longitude (max)	latitude (min)	latitude (max)
SHED7	−124.99	−97.05	37.77	54.01
SHED8	−122.39	−73.53	29	63.41
moose	−117.93418	−94.12167	33.546982	59.241871
petrel	−33.44	48.02	−75.05	−57.04
taxi	−0.15	16.24	39.35	51.46
drifter	−132.04	−122.58	48.24	49.79

In each dataset, the location traces are collected at specific intervals. For each agent, we divide the entire duration of the available data into time windows. The length of a window, *T*_w_, is the same as the base/fundamental data collection interval of the dataset. We assign one sample to each time window. If location accuracy data are available, the most accurate location estimate closest to the start of the time window is chosen. If accuracy information is unavailable, we consider the sample closest to the beginning of the window. When multiple location samples *S* share the most favourable accuracy and timestamp, the sample used is drawn with uniform probability from *S*. The resultant sequences of GPS traces are mapped to spatial grids at different spatial quantization, and further down-sampled using different sampling periods. We generate location sequences at sampling interval *T* and spatial resolution *d* as follows:

*Down-sampling*: We choose every (*T*/*T*_w_)th sample, if available, from the string constructed using time window *T*_w_, as mentioned above. The list of down-sampling intervals are provided in table 2.
*Spatial quantization*: Each record is mapped to a grid based on a record’s distance from the top-left corner of the study bounding box. For a given (*lat, long*), the corresponding row and column numbers in the grid are calculated employing the Haversine distance metric [15]. Following [8], the space covered by the dataset was gridded into cells 4 km across, then down-sampled using a quadtree decomposition to a minimum grid cell size of either 62.5 or 15.625 m, encompassing the accuracy of ranging from that of commodity GPS hardware, as employed in [16], on the fine-grained side, to the transmission range of cell towers in suburban areas, as in [1], on the coarse side.

**Table 2.** Down-sampling intervals of different datasets.

dataset	down-sampling Intervals
SHED7	5 min, 10 min, 30 min, 1 h, 2 h, 4 h, 8 h
SHED8	5 min, 10 min, 30 min, 1 h, 2 h, 4 h, 8 h
taxi	1 min, 5 min, 10 min, 30 min, 1 h, 2 h, 4 h, 8 h
moose	1 h, 2 h, 3 h, 4 h, 5 h, 6 h, 7 h, 8 h
petrel	30 min, 1 h, 2 h, 3 h, 4 h, 5 h, 6 h, 7 h, 8 h
drifter	10 min, 30 min, 1 h, 2 h, 4 h, 8 h

Trajectories through the discretized space were rendered as visit strings. These strings were down-sampled at regular intervals, with a maximum period (inter-sampling interval) of 8 h and a minimum of between 1 min and 1 h, depending on the structure of the data. Trajectory duration was conserved to compare the same paths through time, leading to decreasing *L* with *T*, as longer sampling periods yielded shorter strings for the same trajectories.

Each generated string for each entity in each dataset had its entropy approximated using (2.1), implemented in custom C++ code. Entropies for each dataset at each spatial and temporal resolution were averaged over agents to generate the entropy rate central tendency for each dataset and each spatio-temporal resolution. Assuming that the means are stable under resampling, the summations in (2.16) can be treated as unknown constant parameters multiplying the associated functions of *d*,*T*,*L* in (2.16) (serving as covariates) and regressing dependent value *H*(*d*,*T*) against independent variables *d* and *T*. The summation terms from (2.16) were fit to each entropy rate over (*d*,*T*) for each dataset using Eureqa from Nutonian Inc. [17,18], using absolute error as the optimization metric. Mean squared error (MSE) is reported as a goodness-of-fit metric.

## 3. Results

Changing spatial and temporal resolution changes the distribution of repeated symbols within substrings sampled from a single cell. At the smallest spatial and temporal scales, fine motion is captured, but stationary periods will be strongly represented for regularly immobile agents such as undergraduate students. As the inter-sample interval increases, substrings become increasingly short, until visit strings such as those associated with brief commutes are dropped entirely. Similarly, as spatial bin size increases, longer repeated substrings are expected, accompanied by correspondingly lower entropy rates.

**Figure 1.**
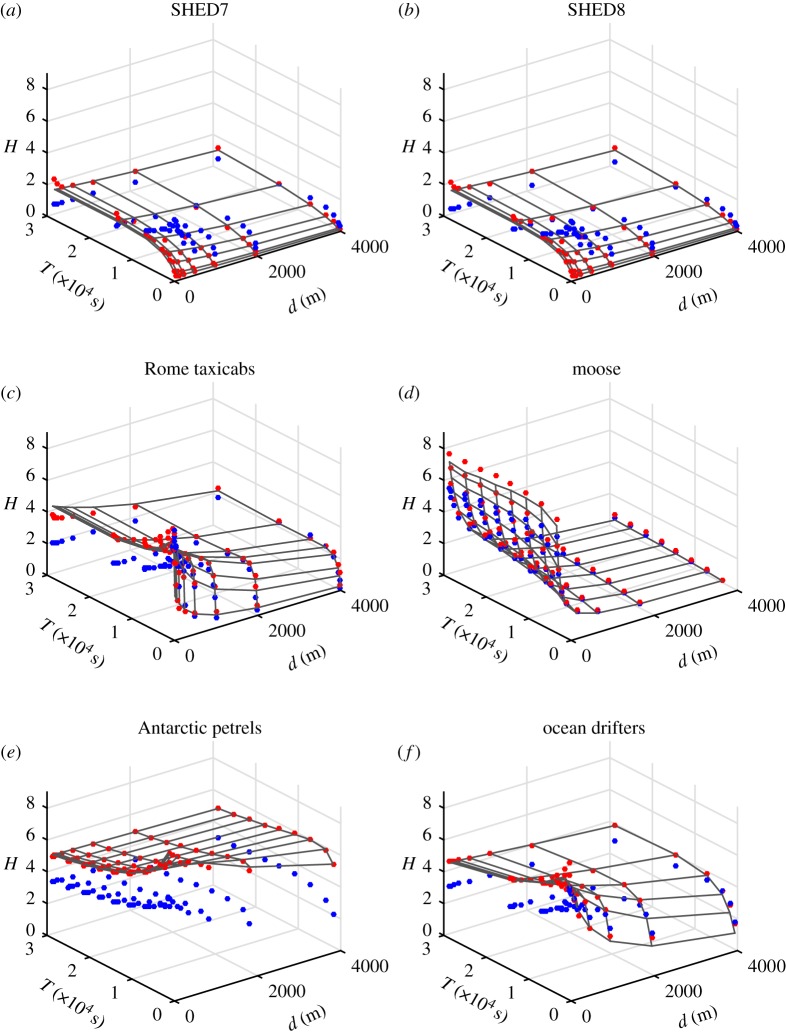
Entropy surface and empirical points for (*a*) university students during summer term, (*b*) university students during autumn term, (*c*) taxicabs in Rome, (*d*) moose in south-central Saskatchewan, (*e*) Antarctic petrels and (*f*) buoys in the Juan de Fuca Strait. *d* is in metres, and *T* in seconds, *H* is in bits. The surface vertices are as given by the entropy of empirical data points, while red data points indicate theory-based estimates using (2.16), and blue points are the calculated entropy rates using the model from [8]. Petrels exhibited the greatest entropy. Notable similarities exist between students, regardless of season, and between taxis and drifters. Moose have unique profiles reflective of their nomadic nature. Departures from predictions of (2.16) are evident in the student datasets for large *d* and small *T*, implying a change in scaling behaviour.

Fitting was able to achieve a strong match between the model and data for most datasets. Mean squared error was less than 10% of the total span of entropy rates calculated empirically. While they offer limited reliability in nonlinear fitting, *R*^2^-values were greater than 0.9. Table 3 summarizes the fit quality and resulting coefficients. Surfaces denoting the model over the range of *d* and *T* values considered, and the calculated entropy rates, are shown in figure 1 using red points, whereas the blue points are the calculated entropy rates using the model from [8]. It is clear that (2.16) provides an accurate description of how mobility entropy rates vary across a wide range of spatial and temporal scales, whereas the model described in [8] diverges from the empirical data in many cases. Because [8] assumed non-overlapping paths, which were traversed at piece-wise constant speeds, datasets where these assumptions were violated show poor fits between model and data. Moose tend to wander over fixed regions browsing, leading to the paths most closely associated with the assumptions in [8], and is well modelled except at small *d*, where entropy increases more sharply than expected. The model from [8] is only valid for human mobility at moderate spatial and temporal scales, and almost always underestimates the entropy rate for petrels and drifters. Unlike the [8] model, (2.16) provides good fits across a wide range of spatial and temporal resolutions for a wide range of path behaviours.

**Table 3.** Constants after fitting (2.16) using nonlinear regression, with *R*^2^ and MSE. Fits are less dependent on squared velocity but show variability in squared dwell time and linear terms.

dataset	$\overset{n}{\underset{i=1}{\sum }}\frac{1}{{v_{i}^{*}}^{2}}$	$\overset{n}{\underset{i=1}{\sum }}{t_{d_{i}}}^{2}$	$\overset{n}{\underset{i=1}{\sum }}\frac{t_{d_{i}}}{v_{i}^{*}}$	$\overset{n}{\underset{i=1}{\sum }}\frac{1}{v_{i}^{*}}$	$\overset{n}{\underset{i=1}{\sum }}t_{d_{i}}$	*R*^2^	*MSE*
SHED7	2.24 × 10^−2^	6.42 × 10^10^	2.59 × 10^7^	1.65 × 10^1^	2.89 × 10^6^	0.93	0.027
SHED8	2.02 × 10^−5^	5.59 × 10^10^	2.13 × 10^7^	4.54 × 10^1^	2.82 × 10^6^	0.95	0.016
taxi	1.63 × 10^−5^	2.75 × 10^8^	4.77 × 10^5^	9.40 × 10^1^	4.34 × 10^5^	0.93	0.1498
moose	1.14 × 10^−1^	4.78 × 10^10^	3.07 × 10^9^	2.47 × 10^4^	2.07 × 10^7^	0.98	0.093
petrel	4.92 × 10^−9^	4.75 × 10^−3^	7.43 × 10^5^	4.53 × 10^−5^	1.12 × 10^6^	0.996	0.005
buoy	1.25 × 10^−1^	8.22 × 10^8^	2.44 × 10^6^	1.05 × 10^1^	9.66 × 10^5^	0.98	0.045

Deviations from the new model exist, particularly for small *d* across all datasets, probably because of the impact of the unmodelled Gaussian noise in the measured locations [8]. Deviation between model and empirical entropy rates are also evident at large *d* and *T*, particular for the moose datasets. The scaling relationship underestimates the entropy rate at large cell sizes and sampling periods, indicating that the sampling of the path parameters has become sufficiently coarse that the summation terms in (2.16) can no longer be treated as constants.

Undergraduate mobility is characterized by relatively low entropies at small *T*, as students are likely to spend prolonged intervals at the university or home, creating highly compressible movement strings containing long runs of repeated location symbols. Taxi drivers are often on the move, and generally have higher entropy rates than students at all resolutions. This finding confirms Song *et al.*’s hypothesis that mobility entropy rate can be used to compare behaviour between populations [1]. While it is common to find an undergrad in the same spatial location over a span of half an hour, the same cannot be said for taxis, and this holds true across a range of spatial scales.

Because at small temporal scales, both students and taxis have a reasonable probability of being in the same location, increasing the inter-sample interval increases the entropy rate by decreasing the number of observed repetitions over the string. Petrels have few repeating substrings, and, therefore, increasing the sampling rate does not change the compressibility of a string—already well represented as a sequence of unique non-repeating symbols—and instead the entropy rate associated with the same paths decreases with rising *T* according to the numerator log*L*, reflecting the decrease of *L* with *T*.

Moose mobility entropy rate is distinct from both humans and birds in that it is almost invariant with analysed sampling rates. Instead, moose mobility entropy rate falls sharply with *d*, probably due to grazing behaviour, where short wanders happen nearly continuously. Once cell size is large enough to incorporate these meandering paths, entropy rate reaches a stable value, at around 500 m. Plateaus in scaling behaviour can indicate spatial or temporal scales at which spatial behaviours become indistinguishable. Entropy rate scaling analysis can provide insight into what characteristic spatial scales are important for populations under study.

The similarity between surfaces in figure 1 can be encapsulated in the values of the marginal path properties. According to (2.16), the values of each marginal property can be described as shown in table 3. The values for 1/*v*_i_^*2^ are substantially lower than the others, even given that the maximum value of *d*^2^ considered is over 1.6 million square metres. Both SHED datasets have similar values for all remaining terms, indicating a degree of similarity. Likewise, the taxi and buoy coefficients are always within an order of magnitude of each other. The petrel dataset is distinct for having a negligible dependence on *t*_*d*_*i*__^2^ and 1/v*_i_, but comparable dependence on *t*_*d*_*i*__/v*_i_ and *t*_*d*_*i*__ as other datasets, reinforcing our hypothesis that the entropy rate scaling is due to the sampling rate.

## 4. Discussion

The amount of information in a set of trajectories is a concise description of the degree of disorder of the motion, and is related to the limit of predictability for that trajectory set [1]. Like many trajectory-based measures, this is contingent on the spatial and temporal resolution of the measurement, which is jointly a function of the marginal path properties across discretized space and the spatial and temporal scales of measurement, according to a well-defined and empirically validated relationship. In the relationship, we used regression to estimate the mean mobility parameters. In the case of more complex variability with *d* and *T*, a semi-empirical approach can be adopted (see electronic supplementary material) at the cost of theoretical rigour.

The scaling behaviour itself summarizes and exposes characteristics of trajectories, particularly across datasets gathered at differing resolutions. From a simple examination of the scaling surfaces, we obtain the following novel findings:

*Taxis are not like students*: Students are less sensitive to changes in spatial and temporal scale than taxis, and have a lower overall entropy, consistent with spending time in class or at home, suggesting that entropy rate and its scaling properties are an appropriate tool for investigating the relative mobility behaviour of human populations.
*Taxis are like drifters*: Taxis and buoys exhibit sensitivity to spatial and temporal resolution at the same scales, probably driven by least-cost paths through a flowing medium.
*Moose movement traces change at scales below 0.25 km^2^*: The sharp increase in mobility entropy rate for moose at fine spatial resolution across a range of sampling periods indicates a difference in observable behaviour at that spatial scale.
*Mobility entropy scaling has limits*: Petrel paths are highly entropic, implying that the observations are at or above the spatio-temporal resolution of their characteristic mobility scale.

The scaling relationship presented here accurately reproduces the mobility entropy rate for a wide variety of agents moving under their own agency or under the influence of complex deterministic systems. However, we have only considered the aggregate entropy rates across all paths and have not considered individual mobile entropy rate, stratified within-subject mobility entropy rate or the probability of predicting the next location under constraints as presented in Smith *et al.* [7], all of which constitute fertile areas for future research. While the scaling relationship provides exceptional fidelity to empirical data over a wide variety of spatial scales, it is implicitly tied to the data through the regression-derived coefficients. However, for the scales and systems measured here, considerable agreement was obtained. The femtosecond behaviour of moose, or light-year binned trajectories of buoys are unlikely to be of scientific interest. However, the behaviour of humans over metres, measured on the order of hours, is of interest, and showed increasing disagreement with the theory in the student datasets. When disagreements with the theory arise, this indicates a potential phase change in observable mobility behaviour, and the scales at which this occurs have phenomenological interest.

The model presented in [8] had a firm theoretical foundation, but made several strong assumptions about the path structure in order to make the derivation tractable. These assumptions were violated in all but one of the datasets examined, leading to poor practical utility for the model. By observing that symbols could be emitted from both transiting and dwelling behaviours, and expressing the time in each cell as a combination of these parameters, we were able to provide good fits across all datasets. However, this came at the cost of model complexity as we moved from the two degree of freedom model (*x* and *u*) in [8] to a three degree of freedom model (*L*, v*_i_, *t*_*d*_*i*__). Additionally, because we could not directly measure v*_i_ and *t*_*d*_*i*__, we were forced to treat the model as a five degree of freedom model to capture the sum of squares. However, this five degree of freedom model provides a theoretically grounded fit for all measured data, lending credibility to our conclusions that this model can be employed to describe entropy rate scaling in mobile systems. We carried forward the assumption of non-overlapping paths in deriving the model (2.16). The modification of the analysis to allow for overlapping paths is not trivial. However, the impact seems to be minimal indicating that either overlapping paths have a limited impact on the entropy rate of physical systems (the incremental savings in compression to an additional symbol is much less than removing repeated sequences) or that the impact has a similar structural form as (2.16), and the effect is absorbed into the fit constants in the summation.

The central theoretical contribution of this work is the demonstrated ability to separate path properties from measurement scale properties in entropy rate calculations. Comparison between students, taxis and buoys has demonstrated that classes of mobility entropy rate are likely to exist, manifested through the social, psychological and physical constraints of the system. When employing this methodology to describe datasets, a vocabulary of mobility classes could emerge, providing further insights.

## 5. Conclusion

Song *et al.* established that mobility entropy rates could characterize the predictability of human mobility traces [19]. This seminal work allowed within-subject comparison of overall path quality, but was limited by the scale dependence of the metric employed. Osgood *et al.* observed that much of the scaling behaviour in mobility entropy rate could be accounted for by examining the structure of visit strings emerging from stylized trajectories [8]. By extending Osgood *et al.*’s work, we were able to obtain a general scaling relationship that has been validated for empirical mobility datasets from students to moose and taxis to drifters, and to describe novel findings about the relative properties of these datasets. While the work here has successfully been applied to complex phenomenon centred on populations with a degree of agency and on complex physical paths, it should be equally valid for systems currently well described using stochastic models, such as financial transactions, fluidic phenomena or particle behaviour.

## Supplementary Material

Supporting Information for Multiscale Entropy Rate Analysis of Complex Mobile Agents

## Data accessibility

The Petrel and Roman Taxicab datasets are freely available online, and can be accessed according to [11,13] respectively. As the SHED data contain sufficient information in the GPS traces to allow reidentification of participants after cross-linking with publicly available information (e.g. phone books, company registries) the data cannot be released in an open fashion. An application for secondary use of the data can be made to the University of Saskatchewan Behavioural Ethics Board, if ethics approval from the hosting institution has been obtained and the corresponding researchers undertake not to cross-link the data with potentially identifiable information. The Moose and Drifter datasets were shared with us by colleagues who have not yet disclosed their data, under the condition that the data not be shared. As we do not own the data, we cannot disclose it.
