# Supplementary material for: Multiscale entropy rate analysis of complex mobile agents
Source: R Soc Open Sci. 2018 Oct 10;5(10):180488. doi: 10.1098/rsos.180488 (PMC6227949; doi:10.1098/rsos.180488)
Supplement: Supporting Information for Multiscale Entropy Rate Analysis of Complex Mobile Agents [file rsos180488supp1.pdf]

Electronic Supplementary Material

for

Multiscale Entropy Rate Analysis of Complex Mobile Agents

Tuhin Paul, Kevin G. Stanley, and Nathaniel D. Osgood

Department of Computer Science, University of Saskatchewan, Saskatoon, SK S7N 5C9, Canada

March 26, 2018

This appendix provides details on the theory and derivation of the model of entropy rate scaling presented in the main paper as well as additional details on the data conditioning employed. A step-by-step derivation of the scaling law is provided. The presence of maxima/minima of the scaling law is discussed, and the behavior of the scaling law is described at the limits of spatio-temporal resolution. This section also provides details on data collection, study population, data pre-processing, and spatio-temporal quantization of the data. The fitting protocols applied for the developed model are also described.

The theoretical model and the dataset details in this section complement the presentation and logic in the main manuscript to provide a clear insight into the internals of the model and how this applies to the comparison of mobility studies performed on different agents and/or at different spatio-temporal resolutions.

1 Theory

For this model, the LZ-based entropy rate, given in (1), is the method used to estimate the entropy rate of the mobility string, as employed by other researchers [11, 10, 9]. Our implementation of (1) is available at `https://github.com/tuhinpaul/lz_entropy_rate`. In (1), L is the length of the sequence and Λ_i is the length of the smallest sub-string that begins at the zero-based index i and was not encountered in positions 0 to $(i-1)$.

$$H = \left(\frac{1}{L} \sum_{i=0}^{L-1} \Lambda_i \right)^{-1} \log L \qquad (1)$$

The theoretical model of the scaling of entropy rate with spatio-temporal resolution, proposed by Osgood *et al.* estimates entropy rate according to (2).

$$H(d,T) = \frac{4\log\frac{x}{\bar{u}T}}{\left(\frac{d}{\bar{u}T}+4\right)} \tag{2}$$

where d is the spatial scale, T is the sampling interval, x is the total travel distance, and $\bar{u}$ is the average velocity [6].

In this work, we extend the theoretical model, and apply it to empirical datasets from a wide variety of sources. The extended model considers the velocity and dwell time of the agents in formulating the entropy rate. Dwell times follow a power-law distribution, and constitute a major part of the real life mobility traces [11, 7].

Let the spatio-temporal resolution be represented as a tuple (T, d), where T is the sampling interval and d is the side length of a square cell in the spatial grid. We assume square cells for simplicity in spatial quantization, and define d as the length of an edge, or the characteristic length of a cell.

The time spent while in motion in the ith cell is expressed as t_{m_i}. Without considering the dwell time, the apparent average velocity in the ith cell along the path of an agent is shown in (3).

$$v_i^* = \frac{d}{t_{m_i}}. \tag{3}$$

The agent may traverse a distance of $k_i d$ in the ith cell where $k_i \in \mathbb{R}^+$. The actual velocity might vary considerably but the average velocity as observed by the experimenter will appear as v_i^*.

The total time spent in the ith cell is the sum of the time spent in motion and the dwell time. The time spent in motion inside the ith cell is $\frac{d}{v_i^*}$ (from (3)). The total time spent in the ith cell is, therefore, expressed as (4).

$$\begin{aligned} t_i &= t_{m_i} + t_{d_i} \\ &= \frac{d}{v_i^*} + t_{d_i} \end{aligned} \tag{4}$$

The total dwell time in the ith cell is the sum of dwells in the ith cell is shown in (5).

$$t_{d_i} = \sum_k t_{d_i}^k \tag{5}$$

Therefore, the observable average velocity considering dwell time, $\tilde{v}_i$, while passing the ith cell, can be expressed as (6).

$$\tilde{v}_i = \frac{d}{t_i} = \frac{d}{\frac{d}{v_i^*} + t_{d_i}}. \tag{6}$$

Let the agent travel through n cells on its entire path, treating repetitions of a cell separately. The number of blocks of repeating strings along the path

would be represented by n. The total time spent in motion on the entire path is expressed as t_m, which is the sum of each t_{m_i}, as shown in (7).

$$t_m = \sum_{i=1}^{n} t_{m_i}. \tag{7}$$

Considering only motion, and nd as the total traversed length, the apparent average velocity v^* is shown in (8).

$$v^* = \frac{nd}{t_m}. \tag{8}$$

The total dwell time along the entire path is the summation of dwell times in each cell, as shown in (9).

$$t_d = \sum_{i=1}^{n} t_{d_i} \tag{9}$$

The observable average velocity for the entire path, considering dwell times as well, is represented as $\tilde{v}$, as shown in (10).

$$\begin{aligned} \tilde{v} &= \frac{nd}{t_1 + t_2 + \ldots + t_n} \\ &= \frac{nd}{(t_{m_1} + t_{d_1}) + (t_{m_2} + t_{d_2}) + \ldots + (t_{m_n} + t_{d_n})} \\ &= \frac{nd}{\sum\limits_{i=1}^{n} t_{m_i} + \sum\limits_{i=1}^{n} t_{d_i}} \end{aligned} \tag{10}$$

From (7), we find that $\sum\limits_{i=1}^{n} t_{m_i} = t_m$. From (8), we find that $t_m = \frac{nd}{v^*}$. Substituting them into (10), we find $\tilde{v}$ as shown in (11).

$$\begin{aligned} \tilde{v} &= \frac{nd}{\sum\limits_{i=1}^{n} t_{m_i} + \sum\limits_{i=1}^{n} t_{d_i}} \\ &= \frac{nd}{t_m + t_d} \\ &= \frac{nd}{\frac{nd}{v^*} + t_d} = \frac{d}{\frac{d}{v^*} + \frac{t_d}{n}}. \end{aligned} \tag{11}$$

There are n blocks along the entire path. Let L_i represent the length of the ith block, and L be the length of the entire string. For simplicity, we assume that L_i is an even integer, and is approximated as (12).

$$L_i = \frac{t_i}{T} = \frac{1}{T}\left(\frac{d}{v_i^*} + t_{d_i}\right). \tag{12}$$

Similarly, L can be expressed as (13):

$$L = \frac{t}{T} = \frac{1}{T}\left(t_m + t_d\right). \tag{13}$$

Assuming a unique terminating symbol at the end of the string representing the path, and using the same decomposition as the theoretical model [6], we can express $\sum_{i=0}^{L-1} \Lambda_i$ in (1) as (14).

$$\begin{aligned}\sum_{i=0}^{L-1} \Lambda_i &= \sum_{i=1}^{n} \sum_{j=1}^{L_i} \Lambda_j \\ &= \sum_{i=1}^{n} \left[2 \sum_{j=1}^{\frac{L_i}{2}} j + \frac{L_i}{2} \right] \\ &= \sum_{i=1}^{n} \left[\frac{L_i^2}{4} + L_i \right].\end{aligned} \tag{14}$$

Substituting (12) into (14), we can express $\sum_{i=0}^{L-1} \Lambda_i$ as (15).

$$\begin{aligned}\sum_{i=0}^{L-1} \Lambda_i &= \sum_{i=1}^{n} \left[\frac{\left(\frac{1}{T}\left(\frac{d}{v_i^*} + t_{d_i}\right)\right)^2}{4} + \frac{1}{T}\left(\frac{d}{v_i^*} + t_{d_i}\right) \right] \\ &= \frac{1}{4T^2} \sum_{i=1}^{n} \left[\left(\frac{d}{v_i^*} + t_{d_i}\right)^2 + 4T\left(\frac{d}{v_i^*} + t_{d_i}\right) \right] \\ &= \frac{1}{4T^2} \sum_{i=1}^{n} \left[d^2 \frac{1}{{v_i^*}^2} + t_{d_i}^2 + 2d\frac{t_{d_i}}{v_i^*} + 4dT\frac{1}{v_i^*} + 4Tt_{d_i} \right] \\ &= \frac{1}{4T^2} \left(d^2 \sum_{i=1}^{n} \frac{1}{{v_i^*}^2} + \sum_{i=1}^{n} t_{d_i}^2 + 2d \sum_{i=1}^{n} \frac{t_{d_i}}{v_i^*} + 4dT \sum_{i=1}^{n} \frac{1}{v_i^*} + 4T \sum_{i=1}^{n} t_{d_i} \right).\end{aligned} \tag{15}$$

The entropy rate $H(d, T)$ from (1) can, therefore, be expressed as (16). Let the summations in (16) be quantified as shown in (17) - (21).

$$C_1 = \sum_{i=1}^{n} \frac{1}{{v_i^*}^2}, \tag{17}$$

$$C_2 = \sum_{i=1}^{n} t_{d_i}^2, \tag{18}$$

$$H(d,T) = \left(\frac{1}{L}\sum_j \Lambda_j\right)^{-1} \log L$$
$$= \frac{4T^2 \log L}{\frac{d^2}{L}\sum_{i=1}^{n}\frac{1}{{v_i^*}^2} + \frac{1}{L}\sum_{i=1}^{n} t_{d_i}^2 + \frac{2d}{L}\sum_{i=1}^{n}\frac{t_{d_i}}{v_i^*} + \frac{4dT}{L}\sum_{i=1}^{n}\frac{1}{v_i^*} + \frac{4T}{L}\sum_{i=1}^{n} t_{d_i}}. \tag{16}$$

$$C_3 = \sum_{i=1}^{n} \frac{t_{d_i}}{v_i^*}, \tag{19}$$

$$C_4 = \sum_{i=1}^{n} \frac{1}{v_i^*}, \tag{20}$$

$$C_5 = \sum_{i=1}^{n} t_{d_i}. \tag{21}$$

Substituting (17) - (21) into (16), we can express $H(d,T)$ as shown in (22).

$$H(d,T) = \left(d^2\frac{C_1}{4T^2L} + \frac{C_2}{4T^2L} + 2d\frac{C_3}{4T^2L} + d\frac{C_4}{TL} + \frac{C_5}{TL}\right)^{-1} \log L. \tag{22}$$

Although t_i values can be approximated from GPS data, t_{m_i}, t_{d_i}, or v_i^* values can not be reliably extracted without additional speed data. We assume that the sums of the terms involving these quantities approximate the true sums within the over distances and sampling rates of interest, or that the values of d and T describe a single regime of the model. At large sampling intervals and kilometer-level spatial quantization, the deviations of the sums become significant, and the model is expected to deviate at those coarse spatio-temporal resolutions.

1.1 Variable Coefficient Analysis

In the main body of this chapter, we argued that C_1 — C_5 could be regarded as independent of d and T if the sums were stable, which was demonstrated to hold true for all datasets for all but the largest values of d and T. Here we extend the analysis to capture how C_1 — C_5 might vary with d and T.

We can express $\frac{C_5}{TL}$ in (22) as (23).

$$\begin{aligned}\frac{C_5}{TL} &= \frac{\sum_{i=1}^{n} t_{d_i}}{T\frac{1}{T}(t_m + t_d)} && \text{From (21) and (13)} \\ &= \frac{t_d}{t_m + t_d} && \text{From (9)}\end{aligned} \tag{23}$$

Therefore, the term $\frac{C_5}{TL}$ corresponds to the fraction of dwell time in the total travel time.

$d\frac{C_4}{TL}$ in (22) can be expressed as (24).

$$\begin{aligned} d\frac{C_4}{TL} &= \frac{\sum_{i=1}^{n} \frac{d}{v_i^*}}{T\frac{1}{T}\left(t_m + t_d\right)} && \text{From (20) and (13),} \\ &= \frac{t_m}{t_m + t_d} && \text{From (3) and (7).} \end{aligned} \tag{24}$$

The quantity $d\frac{C_4}{TL}$ corresponds to the fraction of non-dwell time along the entire path.

$d^2\frac{C_1}{4T^2L} + \frac{C_2}{4T^2L} + 2d\frac{C_3}{4T^2L}$ in (22) can be expressed as (25).

$$\begin{aligned} d^2\frac{C_1}{4T^2L} + \frac{C_2}{4T^2L} + 2d\frac{C_3}{4T^2L} &= d^2\frac{\sum_{i=1}^{n}\frac{1}{v_i^{*2}}}{4T^2L} + \frac{\sum_{i=1}^{n} t_{d_i}^2}{4T^2L} + 2d\frac{\sum_{i=1}^{n}\frac{t_{d_i}}{v_i^*}}{4T^2L} \\ &= \frac{\sum_{i=1}^{n}\frac{d^2}{v_i^{*2}} + \sum_{i=1}^{n} t_{d_i}^2 + \sum_{i=1}^{n} 2\frac{dt_{d_i}}{v_i^*}}{4T^2L} \\ &= \frac{\sum_{i=1}^{n}\left(\frac{d}{v_i^*} + t_{d_i}\right)^2}{4T^2\frac{1}{T}\left(t_m + t_d\right)} && \text{From (13)} \\ &= \frac{\sum_{i=1}^{n} {t_i}^2}{4T\left(t_m + t_d\right)} && \text{From (4)} \\ &= \frac{t}{4T}\sum_{i=1}^{n}\frac{{t_i}^2}{t^2} && \text{From (4).} \end{aligned} \tag{25}$$

In (25), $\frac{t_i}{t}$ is the fraction of the time spent in the ith cell. Let this ratio be expressed as f_i. Then, we can rewrite (25) as (26).

$$\begin{aligned} d^2\frac{C_1}{4T^2L} + \frac{C_2}{4T^2L} + 2d\frac{C_3}{4T^2L} &= \frac{t}{4T}\sum_{i=1}^{n} f_i^2 \\ &= \frac{L}{4}\sum_{i=1}^{n} f_i^2 && \text{From (13).} \end{aligned} \tag{26}$$

By substituting (26) in to (22), $H(d,T)$ can be rewritten as (27).

$$H(d,T) = \left(\frac{L}{4}\sum_{i=1}^{n} f_i^2 + d\frac{C_4}{TL} + \frac{C_5}{TL}\right)^{-1}\log L. \tag{27}$$

If we know the distribution of f_i, we can approximate $\sum_{i=1}^{n} f_i^2$ when n changes due to change in (T, d). By expressing the sum as $f(d,T)$, we can then rewrite $H(d,T)$ from (27) as (28).

$$H(d,T) = \left(\frac{L}{4} f(d,T) + d\frac{C_4}{TL} + \frac{C_5}{TL}\right)^{-1} \log L. \tag{28}$$

We can rely on empirical methods to estimate $f(d,T)$. Using Eureqa [3], we empirically found a solution for $f(d,T)$ as shown in (29), which works in general form across datasets:

$$f(d,T) = C_6 + C_7 * d + C_8 * T. \tag{29}$$

By substituting (29) into (28), we can, therefore, express $H(d,T)$ as (30).

$$H(d,T) = \left(\frac{L}{4}\left(C_6 + C_7 * d + C_8 * T\right) + d\frac{C_4}{TL} + \frac{C_5}{TL}\right)^{-1} \log L. \tag{30}$$

1.2 Scaling Law Behavior

Knowledge of maxima/minima of the entropy rate for a particular d or T may be useful in the design and evaluation of a mobility study to assess extreme values of the entropy rate. Similarly, the behavior of the model at the limits of d and T may ensure that if the model conforms to the desirable behaviors governed by the structure of the location string at those limits.

Maxima/Minima: Note that, L is a function of T. Substituting (13) into (22), we get (31).

$$H(d,T) = \frac{4Tt \log t - 4Tt \log T}{d^2 C_1 + C_2 + 2dC_3 + 4dTC_4 + 4TC_5}. \tag{31}$$

To check if $H(d,T)$ has a maxima/minima at any d or T, we need to differentiate (22) or (31) with respect to d and T. We find the relation in (32) by differentiating (22) with respect to d. However, because $C_1, \ldots, C_5$ are positive numbers, no practical d or T can be found from (32), as all roots with respect to d and T are negative.

$$T = -\frac{2\left(C_1 d + C_3\right)}{C_4} \tag{32}$$

For convenience, we use (31) to differentiate $H(d,T)$ with respect to T, and find the relation in (33) dictating the presence of maxima/minima:

Given C_1 — C_5, for a given d, (33) can be solved using numerical analysis to find the T pertaining to a maxima/minima of $H(d,T)$.

$$
\begin{aligned}
&\frac{T}{\log\frac{t}{T}-1} = \frac{C_1d+2C_3d+2C_2}{C_4d+C_5}\\
&\implies \frac{T}{\log t-\log T-1} = \frac{C_1d+2C_3d+2C_2}{C_4d+C_5}\\
&\implies T = (\log t-1-\log T)\,\frac{C_1d+2C_3d+2C_2}{C_4d+C_5}\\
&\implies T = (\log t-1)\,\frac{C_1d+2C_3d+2C_2}{C_4d+C_5} - \log T\frac{C_1d+2C_3d+2C_2}{C_4d+C_5}\\
&\implies T + \log T\frac{C_1d+2C_3d+2C_2}{C_4d+C_5} = (\log t-1)\,\frac{C_1d+2C_3d+2C_2}{C_4d+C_5}.
\end{aligned} \tag{33}
$$

Behavior of $H(d,T)$ at Limits: We examine if the model has desirable behavior at the limits of T and d. Behavior at the limits would explain whether the model conforms to theoretical constraints. For a constant T, dictionary size grows as d approaches 0. In the limiting case, all repetitions will be the result of dwelling, where C_2 and C_5, from (18) and (21) respectively, pertain to the dwelling of the agent. The repetition from dwelling scales the maximum entropy, $\log L$, of L symbols as follows:

$$
\begin{aligned}
\lim_{d\to 0} H(d,T) &= \lim_{d\to 0} \frac{4T^2L\log L}{d^2C_1+C_2+2dC_3+4dTC_4+4TC_5} \qquad \text{From (22)}\\
&= \frac{4T^2L\log L}{C_2+4TC_5}
\end{aligned} \tag{34}
$$

When the cell size is very large ($d\to\infty$), all samples fall into the same cell, resulting in zero entropy rate, which is independent of the temporal resolution. From (22), this is mathematically presented as (34).

$$
\begin{aligned}
\lim_{d\to\infty} H(d,T) &= \lim_{d\to\infty} \frac{4T^2L\log L}{d^2C_1+C_2+2dC_3+4dTC_4+4TC_5}\\
&= 0.
\end{aligned} \tag{35}
$$

For convenience, we use (31) to find the entropy rates at the limits of T. When d is a constant and $T\to 0$, entropy rate goes to zero, as shown in (36), because we end up with longer and longer strings of repeating locations with high compressibility:

$$
\begin{aligned}
\lim_{T\to 0} H(d,T) &= \lim_{T\to 0} \frac{4Tt\log t-4Tt\log T}{d^2C_1+C_2+2dC_3+4dTC_4+4TC_5}\\
&= 0.
\end{aligned} \tag{36}
$$

When d is a constant and $T \to \infty$, then entropy rate is undefined as shown in (37).

$$\begin{aligned}
\lim_{T\to\infty} H(d,T) &= \lim_{T\to\infty} \frac{4Tt\log t - 4Tt\log T}{d^2C_1 + C_2 + 2dC_3 + 4dTC_4 + 4TC_5} \\
&= \lim_{T\to\infty} \frac{\frac{4Tt\log t}{T\log T} - \frac{4Tt\log T}{T\log T}}{\frac{d^2C_1+C_2+2dC_3+4dTC_4+4TC_5}{T\log T}} \\
&= \lim_{T\to\infty} \frac{\frac{4t\log t}{\log T} - 4t}{\frac{d^2C_1+C_2+2dC_3+4dTC_4+4TC_5}{T\log T}} \\
&= \text{undefined.}
\end{aligned} \tag{37}$$

This is sensible because when $T \to \infty$, we can not have enough samples to evaluate $H(d,T)$ at that T for varying d.

We can also consider how the model behaves at extreme values of speed and dwell times. If $\forall_i\ t_{d_i} \to 0$, then entropy rate should depend on apparent average velocities at each cell and dwell times do not effect the entropy rate. The model conforms to this case of motion without dwelling, as shown in (38).

$$\begin{aligned}
\lim_{\forall_i\ t_{d_i}\to 0} H(d,T) &= \lim_{\forall_i\ t_{d_i}\to 0} \frac{4T^2\log L}{\frac{d^2}{L}\sum_{i=1}^{n}\frac{1}{{v_i^*}^2} + \frac{1}{L}\sum_{i=1}^{n} t_{d_i}^2 + \frac{2d}{L}\sum_{i=1}^{n}\frac{t_{d_i}}{v_i^*} + \frac{4dT}{L}\sum_{i=1}^{n}\frac{1}{v_i^*} + \frac{4T}{L}\sum_{i=1}^{n} t_{d_i}} \\
&= \lim_{\forall_i\ t_{d_i}\to 0} \frac{4T^2\log L}{\frac{d^2}{L}\sum_{i=1}^{n}\frac{1}{{v_i^*}^2} + \frac{4dT}{L}\sum_{i=1}^{n}\frac{1}{v_i^*}}.
\end{aligned} \tag{38}$$

However, if the dwell time at the jth cell approaches ∞, all samples after reaching that cell will be the same. The apparent average speed within the cell would approach 0. Because the cell would get stuck in the jth cell, the time in that cell and the length of the substring emanating from that cell will be determined by the dwell time in that cell considering total observation time. The dwell time in the jth block, in this case, is t_{d_j} as shown in (39), and the length of the jth block is L_j as shown in (40).

$$t_{d_j} = t - \sum_{i=1}^{j-1} t_i \tag{39}$$

$$L_j = \frac{t_{d_j}}{T} = \frac{t - \sum_{i=1}^{j-1} t_i}{T} \tag{40}$$

Considering (14) for the jth block, the entropy rate can be expressed as (41).

$$\lim_{\exists_j \ t_{d_j} \to \infty} H(d,T) = \lim_{\exists_j \ t_{d_j} \to \infty} \frac{4T^2 \log L}{\frac{d^2}{L}\sum_{i=1}^{n}\frac{1}{v_i^{*2}} + \frac{1}{L}\sum_{i=1}^{n} t_{d_i}^2 + \frac{2d}{L}\sum_{i=1}^{n}\frac{t_{d_i}}{v_i^*} + \frac{4dT}{L}\sum_{i=1}^{n}\frac{1}{v_i^*} + \frac{4T}{L}\sum_{i=1}^{n} t_{d_i}}$$
$$= \frac{4T^2 \log L}{\frac{d^2}{L}\sum_{i=1}^{j-1}\frac{1}{v_i^{*2}} + \frac{1}{L}\sum_{i=1}^{j} t_{d_i}^2 + \frac{2d}{L}\sum_{i=1}^{j-1}\frac{t_{d_i}}{v_i^*} + \frac{4dT}{L}\sum_{i=1}^{j-1}\frac{1}{v_i^*} + \frac{4T}{L}\sum_{i=1}^{j} t_{d_i}}. \quad (41)$$

If the agent is observed for infinite time, the entropy rate according to (41) would approach 0.

If the apparent average velocities in the cells approaches ∞, then v_i^* values would not effect the entropy rate in practice and the entropy rate would depend on dwell times:

$$\lim_{\forall_i \ v_i^* \to \infty} H(d,T) = \lim_{\forall_i \ v_i^* \to \infty} \frac{4T^2 \log L}{\frac{d^2}{L}\sum_{i=1}^{n}\frac{1}{v_i^{*2}} + \frac{1}{L}\sum_{i=1}^{n} t_{d_i}^2 + \frac{2d}{L}\sum_{i=1}^{n}\frac{t_{d_i}}{v_i^*} + \frac{4dT}{L}\sum_{i=1}^{n}\frac{1}{v_i^*} + \frac{4T}{L}\sum_{i=1}^{n} t_{d_i}}$$
$$= \frac{4T^2 \log L}{\frac{1}{L}\sum_{i=1}^{n} t_{d_i}^2 + \frac{4T}{L}\sum_{i=1}^{n} t_{d_i}}. \quad (42)$$

2 Data Collection and Features

We used six empirical datasets encompassing mobility of humans [12], taxi cabs [2], animals [5], [13], and ocean drifters [8] to evaluate the performance of the theoretical model. Human mobility patters were taken from the Saskatchewan Human Ethology Datasets (specifically, SHED7 and SHED8) [12], which are linked to the ongoing development of iEpi [4]. The SHED datasets contain detailed mobility, activity, and contact traces from university students and staff. We considered the mobility traces from the taxi cab mobility study conducted by Bracciale *et al.* in Rome, Italy [2] as a contrasting human mobility dataset. Understanding the taxi trace patterns can play an important role as a contrast to the patterns of undergraduates. Taxi cab traces incorporate mobility traces of random people, and are expected to encounter popular routes and important urban locations. We also consider the mobility patterns of wild animals, less constrained by urban enviornments. We assessed the model with the GPS traces that were collected from collars mounted on moose [5] and Antarctic petrels [13]. Because the scaling law does not require movements with agency, we also use the

mobility tracks of ocean surface drifters [8] to validate that the model applies in general to complex physical pheonmenon as well. The drifter data also includes times spent on the land before deployment and if the drifter runs aground prior to retrieval.

The durations of the studies behind the datasets vary largely, as shown in Table 1. The mentioned duration of SHED7 and SHED8, taxi cab, and ocean drifter studies in Table 1 are based on all the available date values in the datasets. The traces in the taxi cab study were sampled at much smaller intervals than in other datasets. Therefore, we limited the location traces to the first fifteen days of the study to make entropy rate calculation feasible within a reasonable amount of time for small T values. For the moose dataset, records between Jan 2012 and Feb 2015 are considered, and this is reflected in Table 1. The records in the petrel dataset span from Dec 2011 to Jan 2014.

The agents/participants of each dataset were passed through a filtering process to ensure that they had a minimum number of records at large sampling intervals.

The base interval in Table 1 refers to the approximate interval of data collection, as we observed in the data or was available from the corresponding study.

Table 1: Dataset details

dataset	SHED7	SHED8	Taxi	Moose	Petrel	Drifter
Duration (days)	35	29	30	1143	777	130
#(Agents)	63	75	316	36	124	9
#(Accepted agents)	56	70	59	36	124	9
Accuracy available?	Y	Y	N	N	N	N
Base Interval	5 min	5 min	15 s	1 hr	30 min	10 min

2.1 Dispersion Maps

Fig. 1 - Fig. 6 show the dispersion of the agents/participants of the datasets, over three days, as heat maps of visited locations. All locations visited by all participants on the selected days are considered. For a given spatial quantization, all locations within a spatial bin were grouped together. The quantization process is described in Data Mediation (Section 2.2). All the locations in a group were represented by the location (latitude, longitude) = $(lat_{group}, lon_{group})$ as follows:

$$lat_{group} = \frac{\text{min(latitude in group)} + \text{max(latitude in group)}}{2} \tag{43}$$

$$lon_{group} = \frac{\text{min(longitude in group)} + \text{max(longitude in group)}}{2} \tag{44}$$

The center and zoom level were set manually to make the presentation legible because otherwise the maps covered larger areas, zooming out the locations of interest. The manual selection of the map center and zoom level dropped out some visited locations - a trade-off made to make the maps comprehensible. Based on the frequency of visits, the visited locations are colored using a gradient from red (most visited) to green (least visited). The scale bars on the bottom-left corners of the maps indicate different distances because of the variation in the speed and span of movement of the corresponding agents. In the SHED (Fig. 1 and Fig. 2) and taxi (Fig. 3) studies, all participants take part in the study at or around the same time. Locations of agents in other datasets may not overlap. We find that SHED participants (Fig. 1 and Fig. 2) visit similar locations from day to day and show different behaviors over the weekend. Similar trends are found in the taxi dataset (Fig. 3) but their dispersion varies significantly based on spatio-temporal resolution. Moose (Fig. 4) visit locations within a larger home range with a few hotspots. Ocean drifters (Fig. 6) change their place significantly from day to day. Similar behaviors are observed in petrels (Fig. 5). Changing spatio-temporal resolutions redistribute hot spots in the maps because of movement of agents to new places. The change in hotspots is less pronounced in humans and moose than in other datasets, indicating their relatively slower movement. Change of hotspots in the taxi dataset is well pronounced. The change of locations of petrel and ocean dirfters are slightly obscured by the relatively large span of locations visited by these agents.

2.2 Data Mediation

For SHED 7, SHED 8, and taxi datasets, we accepted participants having at least fifteen location records (arbitrarily decided) for the largest sampling interval used for the corresponding dataset. For other datasets, we only discarded erroneous location records as described above.

3 Fitting Protocols

We used the Eureqa software [1, 3] to derive the constant terms in our model, shown in (22), from the location sequences after spatio-temporal quantization. Eureqa [3] is an artificial intelligence-based data non-linear regression tool, which estimated the parameters in (22) via global-optimization based nonlinear regression. The input to Eureqa for data regression is a set of $(dset, T, d, L, lzH)$ tuples where lzH is the aggregate entropy rate for the data set $dset$ at spatio-temporal quantization (T, d) and L is the corresponding average sequence length. We used R^2-based goodness of fit and mean squared error as the error metrics to evaluate fit performance.

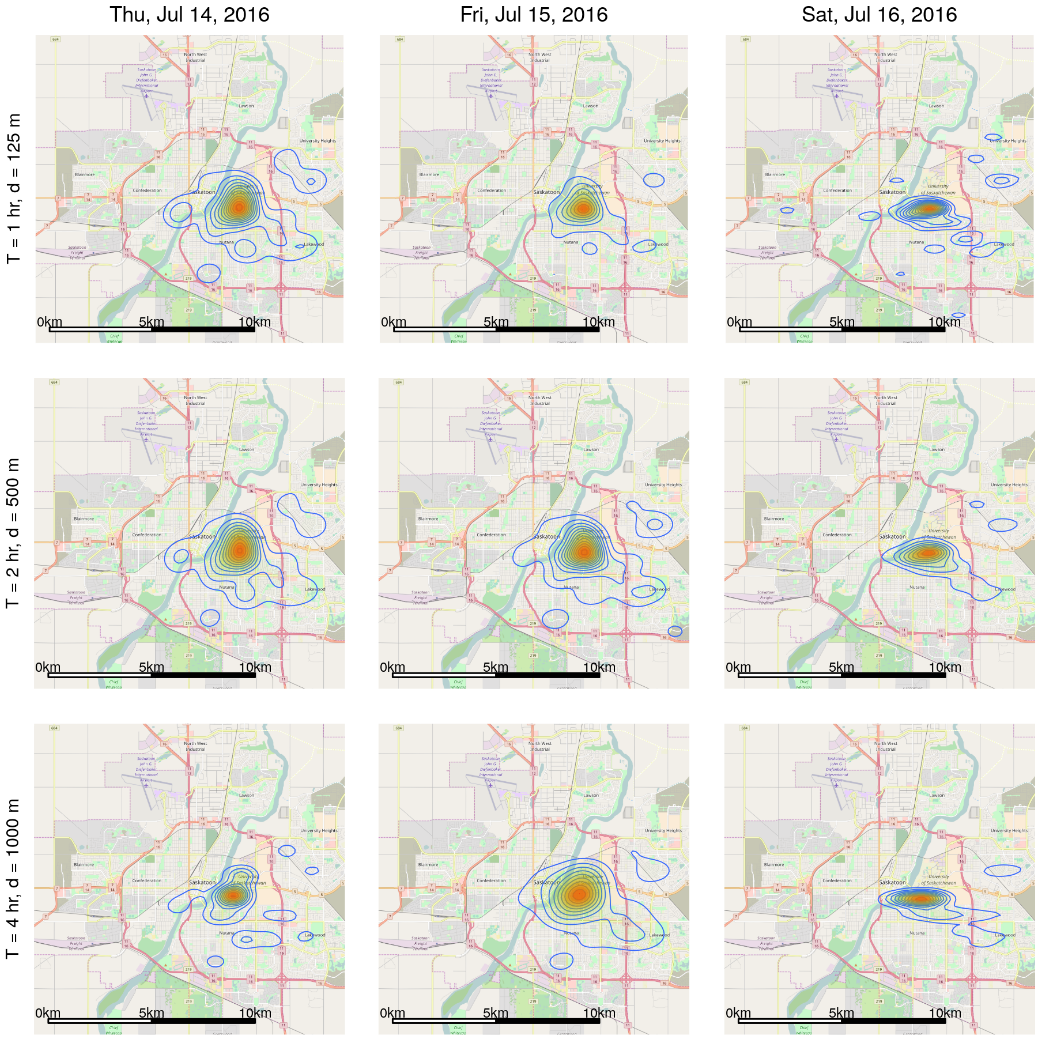

Figure 1: Heatmap of the dispersion of participants (undergraduate students) of SHED 7 over three consecutive days in the summer of 2016. Each column represents a day and each row represents a (T, d) pair. The participants visited fairly similar locations, which were centered around the University of Saskatchewan. Dispersion in the weekend is different than the weekdays. Weekdays exhibit visually similar dispersion.

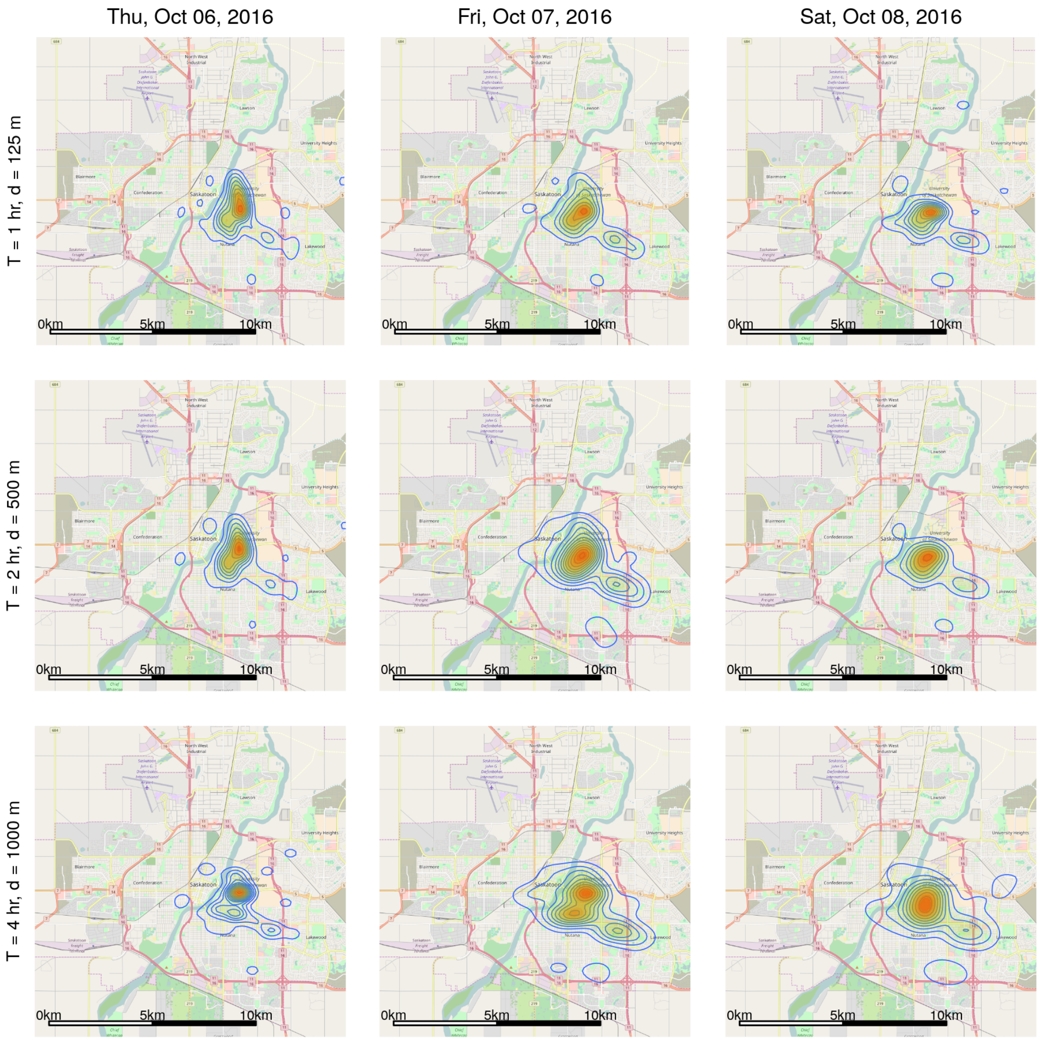

Figure 2: Heatmap of the dispersion of participants (undergraduate students) of SHED 8 over three consecutive days in the fall of 2016. Each column represents a day and each row represents a (T, d) pair. The participants visited fairly similar locations, which were centered around the University of Saskatchewan. Dispersion in the weekend is slightly different than the weekdays. Weekdays exhibit visually similar dispersion. The data exhibit visually less dispersion changes than the summer data in Fig. 1.

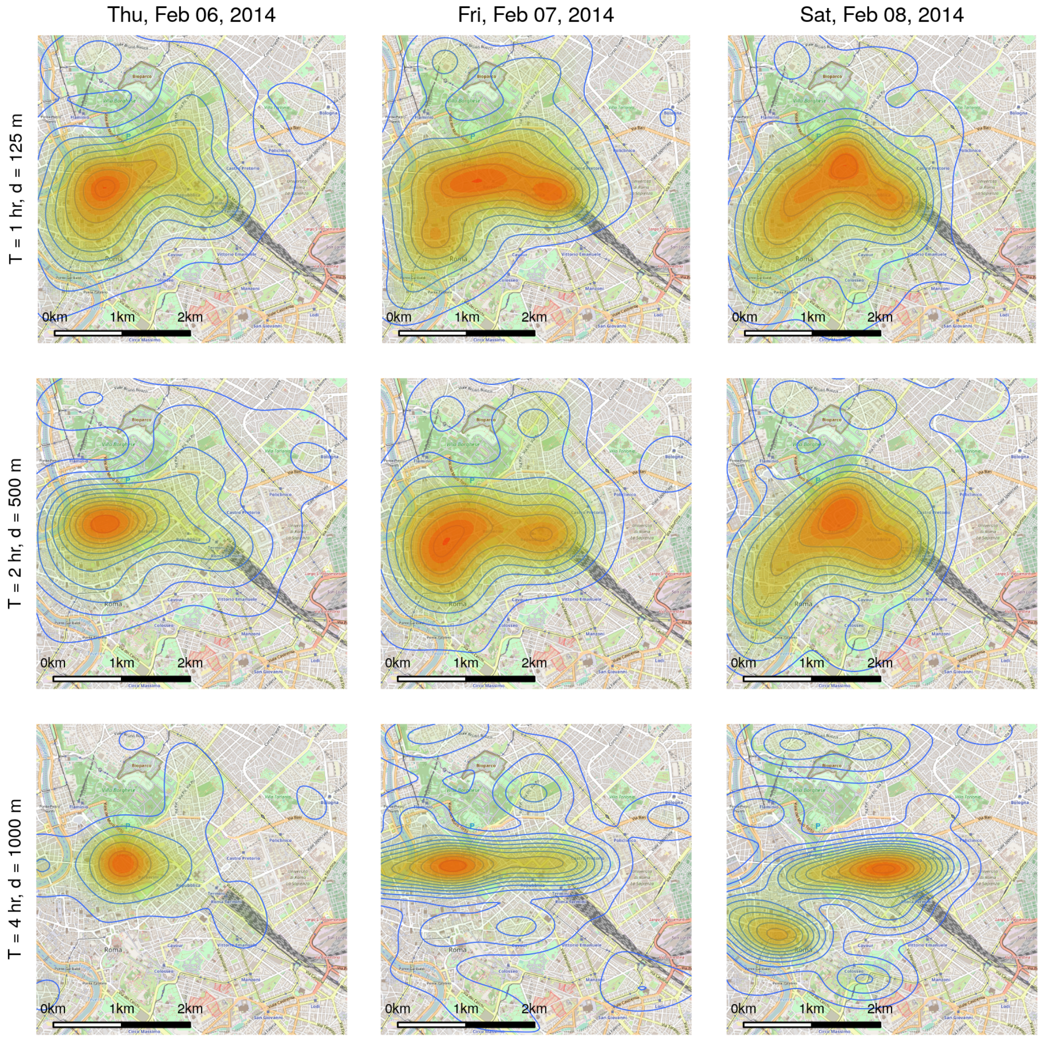

Figure 3: Heatmap of the dispersion of taxi cabs tracked in Rome over three consecutive days. The map area is much smaller than the maps shown for undergraduate students in Fig. 1 and Fig. 2. Taxicabs demonstrate aggregate human movement behaviors. In the weekday, the locations are more concentrated to a hotspot but two hotspots are visible in the weekend.

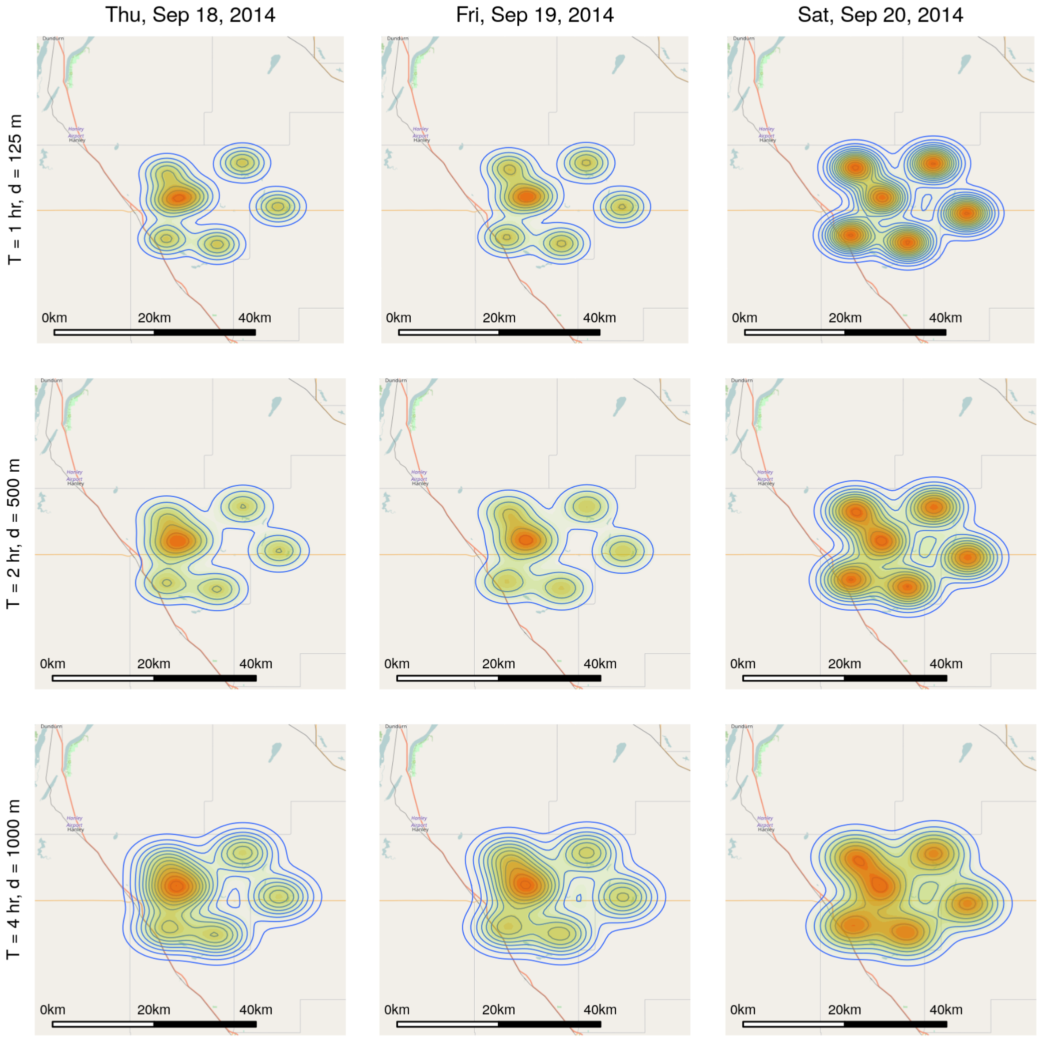

Figure 4: Heatmap of the dispersion of the tracked moose over three consecutive days. The hotspots appear visually stable, which indicate steady grazing behavior.

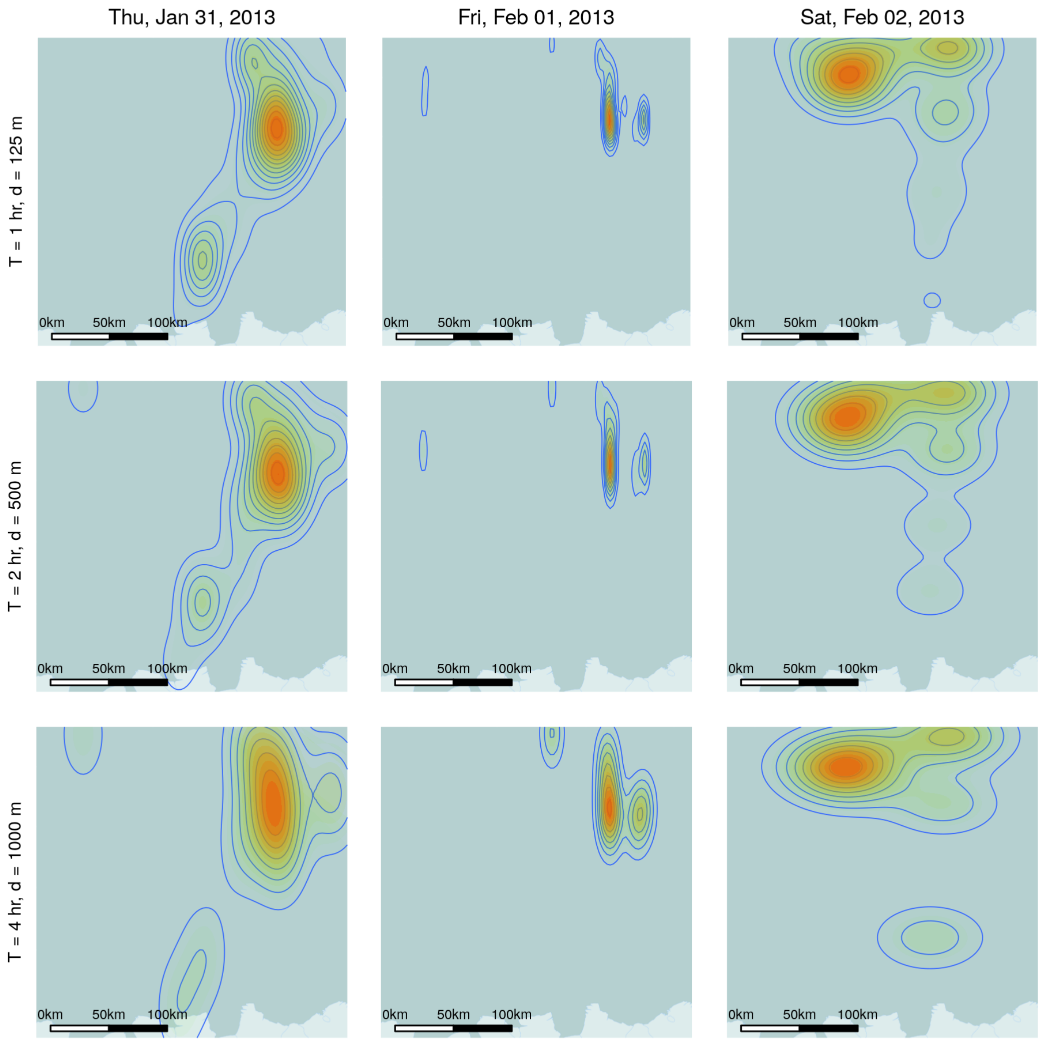

Figure 5: Heatmap of the dispersion of Antarctic Petrels over three consecutive days. Their locations change largely from day to day because petrels fly over wide areas and do not stick to specific locations for long.

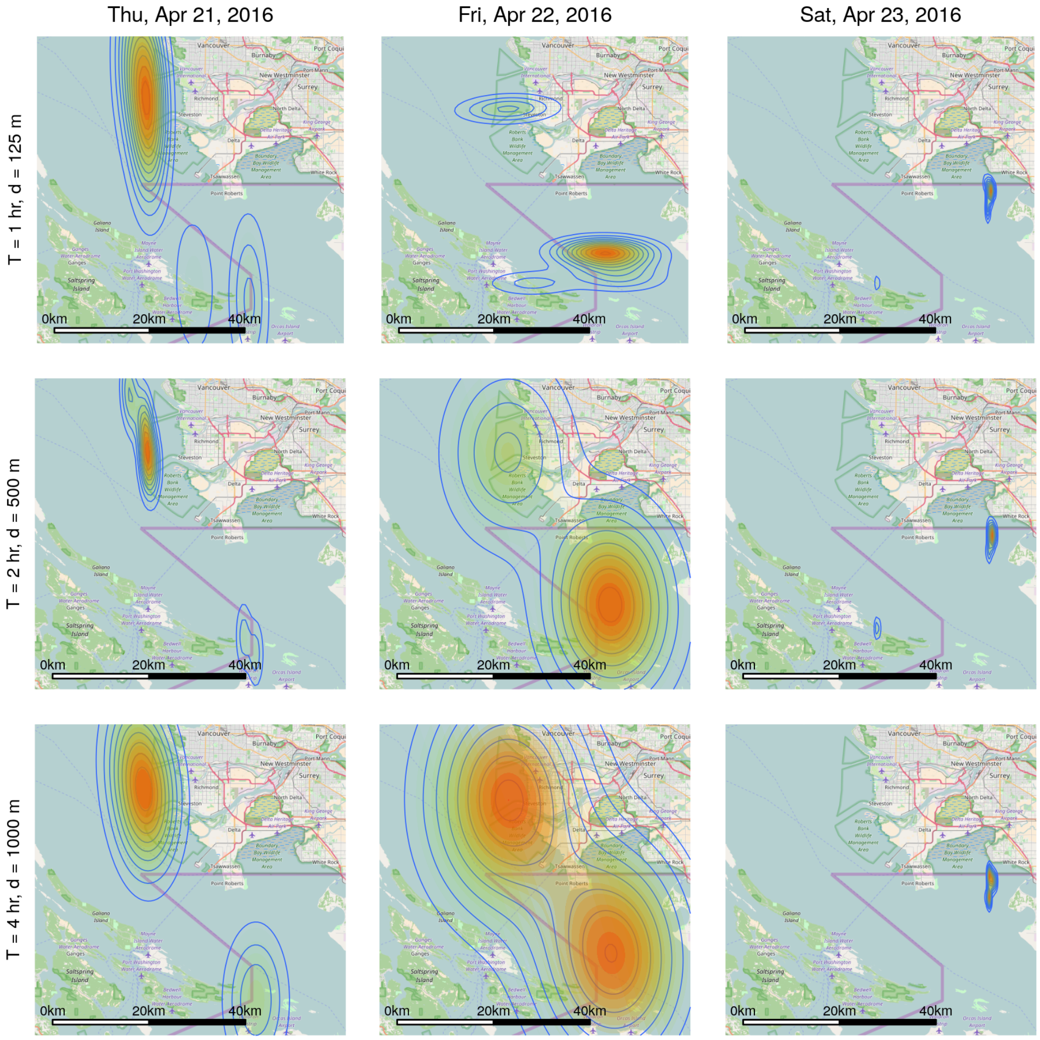

Figure 6: Heatmap of the dispersion of ocean drifters over three consecutive days. Similar to petrels, ocean drifters move to different places due to sea currents as time passes, and show no ties to specific locations.

References

[1] Aryadoust, V. Application of Evolutionary Algorithm-based Symbolic Regression to Language Assessment: Toward Nonlinear Modeling. *Psychological Test and Assessment Modeling 57*, 3 (2015), 301–337.

[2] Bracciale, L., Bonola, M., Loreti, P., Bianchi, G., Amici, R., and Rabuffi, A. CRAWDAD Dataset Roma/taxi (v. 2014-07-17). Downloaded from `http://crawdad.org/roma/taxi/20140717`, July 2014.

[3] Dubčáková, R. Eureqa: Software Review. *Genetic programming and evolvable machines 12*, 2 (2011), 173–178.

[4] Knowles, D. L., Stanley, K. G., and Osgood, N. D. A Field-validated Architecture for the Collection of Health-relevant Behavioural Data. In *2014 IEEE International Conference on Healthcare Informatics (ICHI)* (2014), pp. 79–88.

[5] Laforge, M. P., Michel, N. L., Wheeler, A. L., and Brook, R. K. Habitat Selection by Female Moose in the Canadian Prairie Ecozone. *The Journal of Wildlife Management 80*, 6 (2016), 1059–1068.

[6] Osgood, N. D., Paul, T., Stanley, K. G., and Qian, W. A Theoretical Basis for Entropy-Scaling Effects in Human Mobility Patterns. *PLoS ONE 11*, 8 (2016), 1–21.

[7] Paul, T., Stanley, K., Osgood, N., Bell, S., and Muhajarine, N. Scaling Behavior of Human Mobility Distributions. In *International Conference on Geographic Information Science* (2016), pp. 145–159.

[8] Pawlowicz, R. Monitoring the fraser river fresh water. the drifter project. `http://www.drifters.eos.ubc.ca/drifter-project`. Chief Scientist: Prof Rich Pawlowicz, University of British Columbia, 6339 Stores Rd., Vancouver, BC CANADA V6T 1Z4. Accessed: 2018-03-13.

[9] Rodriguez-Carrion, A., Garcia-Rubio, C., Campo, C., and Das, S. K. Analysis of a Fast LZ-Based Entropy Estimator for Mobility Data. In *IEEE International Conference on Pervasive Computing and Communication Workshops* (2015), pp. 451–456.

[10] Smith, G., Wieser, R., Goulding, J., and Barrack, D. A Refined Limit on the Predictability of Human Mobility. In *IEEE International Conference on Pervasive Computing and Communications (PerCom)* (2014), pp. 88–94.

[11] Song, C., Koren, T., Wang, P., and Barabási, A.-L. Modelling the Scaling Properties of Human Mobility. *Nature Physics 6*, 10 (2010), 818–823.

[12] Stanley, K., Bell, S., Kreuger, L. K., Bhowmik, P., Shojaati, N., Elliott, A., and Osgood, N. D. Opportunistic Natural Experiments Using Digital Telemetry: a Transit Disruption Case Study. *International Journal of Geographical Information Science* (2016), 1–20.

[13] Tarroux, A., Weimerskirch, H., Wang, S.-H., Bromwich, D. H., Cherel, Y., Kato, A., Ropert-Coudert, Y., Varpe, Ø., Yoccoz, N. G., and Descamps, S. Flexible Flight Response to Challenging Wind Conditions in a Commuting Antarctic Seabird: Do You Catch the Drift? *Animal Behaviour 113* (2016), 99–112.
